# Body composition changes in physically active individuals consuming ketogenic diets: a systematic review

**DOI:** 10.1186/s12970-021-00440-6

**Published:** 2021-06-05

**Authors:** Julie L. Coleman, Christopher T. Carrigan, Lee M. Margolis

**Affiliations:** 1 U.S. Army Research Institute of Environmental Medicine, 10 General Greene Ave, Building 42, Natick, MA 01760 USA; 2 Oak Ridge Institute of Science and Education, Belcamp, MD USA

**Keywords:** Ketosis, Fat oxidation, Carbohydrate oxidation, Low carbohydrate, High-fat

## Abstract

**Background:**

To achieve ideal strength/power to mass ratio, athletes may attempt to lower body mass through reductions in fat mass (FM), while maintaining or increasing fat-free mass (FFM) by manipulating their training regimens and diets. Emerging evidence suggests that consumption of high-fat, ketogenic diets (KD) may be advantageous for reducing body mass and FM, while retaining FFM.

**Methods:**

A systematic review of the literature was conducted using PubMed and Cochrane Library databases to compare the effects of KD versus control diets (CON) on body mass and composition in physically active populations. Randomized and non-randomized studies were included if participants were healthy (free of chronic disease), physically active men or women age ≥ 18 years consuming KD (< 50 g carbohydrate/d or serum or whole blood β-hydroxybutyrate (βhb) > 0.5 mmol/L) for ≥14 days.

**Results:**

Thirteen studies (9 parallel and 4 crossover/longitudinal) that met the inclusion criteria were identified. Aggregated results from the 13 identified studies show body mass decreased 2.7 kg in KD and increased 0.3 kg in CON. FM decreased by 2.3 kg in KD and 0.3 kg in CON. FFM decreased by 0.3 kg in KD and increased 0.7 kg in CON. Estimated energy balance based on changes in body composition was − 339 kcal/d in KD and 5 kcal/d in CON. Risk of bias identified some concern of bias primarily due to studies which allowed participants to self-select diet intervention groups, as well as inability to blind participants to the study intervention, and/or longitudinal study design.

**Conclusion:**

KD can promote mobilization of fat stores to reduce FM while retaining FFM. However, there is variance in results of FFM across studies and some risk-of-bias in the current literature that is discussed in this systematic review.

**Supplementary Information:**

The online version contains supplementary material available at 10.1186/s12970-021-00440-6.

## Introduction

Achieving and maintaining body composition associated with optimal performance is an important but difficult goal to meet for many athletes, coaches, trainers, and dietitians [1]. Ideal body composition ranges drastically between sports and individual athletes. While some athletes aim to achieve greater absolute mass to increase total strength/power, athletes competing in sports such as running/cycling, sports with weight classes (e.g., boxing, mixed martial arts, and wrestling), or aesthetic sports (e.g., diving, gymnastics, figure skating, and ballet) need to prioritize achieving an optimal strength/power to mass ratio [2, 3]. To achieve optimal strength/power to mass ratio, those athletes typically aim to achieve a low body mass, through reductions in fat mass (FM), while maintaining or increasing fat-free mass (FFM). To reduce total body mass, athletes may restrict energy intake, resulting in a negative energy balance (e.g., energy expenditure > energy intake) [4, 5]. However, periods of negative energy balance also typically reduce FFM, and that loss of FFM during energy deficit can account for as much as ~ 50% of total mass lost in lean individuals [6–8]. Reductions in FFM during negative energy balance may impair physical performance [9].

Manipulation of macronutrient intake can mitigate loss of FFM during energy deficit and loss of total mass [10]. Recent evidence suggests that a high-fat, ketogenic diet (KD) may reduce total body mass and FM, while retaining FFM [11]. KD are very low in carbohydrate (< 50 g/d), high in fat (60–80% total kcal) and moderate to high in protein (~ 1.2–2.0 g/kg/d) [12]. Adherence to a KD promotes endogenous fat oxidation and increases circulating concentrations of ketone bodies [13, 14] that may contribute to preferential mobilization of fat stores resulting in reduced FM [11, 15, 16]. Additionally, the amount of protein consumed with a KD, roughly twice the recommended dietary allowance (RDA), has been shown to mitigate declines in FFM during weight loss [10]. To date, the majority of studies assessing the impact of KD on body mass and composition have studied overweight and obese sedentary populations [17], precluding translations to physically active populations.

Over the last several years there has been a resurgence in the interest of KD on athletic performance, due to drastic shifts of increased fat oxidation and decreased reliance on carbohydrate as a fuel during exercise [18]. Increased popularity of KD in athletic populations has resulted in a growing body of evidence regarding changes in body mass and composition in physically active individuals adhering to KD [11, 16]. The objective of this systematic review was to aggregate data from multiple investigations to characterize the overall effects of KD on body mass, FM, and FFM in physically active populations.

## Methods

### Literature search strategy

**Fig. 1:**
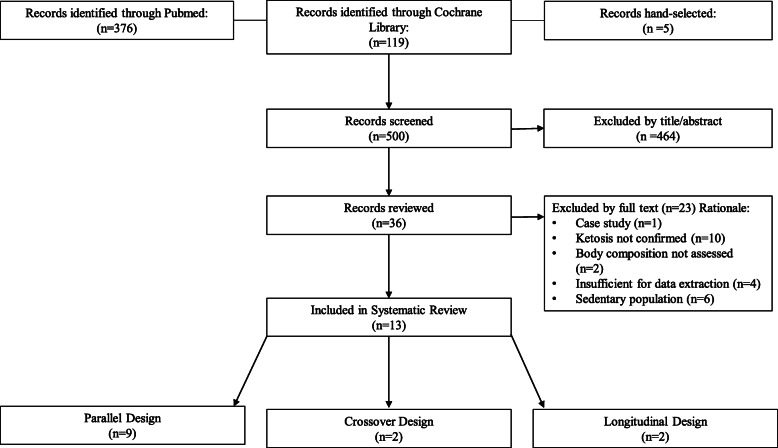
PRISMA search strategy

Abstracts of publications identified in Pubmed (https://www.ncbi.nlm.nih.gov/pubmed/) and Cochrane Library (https://www.cochranelibrary.com/) were organized and reviewed for relevance using the Abstrackr citation program (http://abstrackr.cebm.brown.edu) [19] by two researchers (CTC and JLC). Searches of all identified terms took place on 20 April 2020 and were not restricted by publication date. Additional searches was conducted on 28 December 2020 and 11 March 2021 to confirm no relevant manuscript were published since the time of our initial search. Search terms are presented in Supplementary Table 1. The Preferred Reporting Items for Systematic Reviews and Meta-Analyses (PRISMA) search strategy and further reference narrowing is described in Fig. 1 [20]. Reference lists from these publications were hand-searched for any reports missed by database searches. Relevant studies that were published after the initial searches were assessed for inclusion in the current analysis. One manuscript was hand-selected. Only manuscripts published in English were included in this systematic review. Full-text publications were reviewed for relevance independently by two researchers (CTC and JLC) for KD consumption and body composition outcomes. Discrepancies between researchers were assessed by a third investigator (LMM). The final 13 publications were reviewed by all involved researchers. Search strategy details can be found at the University of York Centre for Reviews and Dissemination (PROSPERO) website (https://www.crd.york.ac.uk/prospero/display_record.php?RecordID=177750).

### Inclusion criteria

Randomized and nonrandomized crossover/longitudinal or parallel controlled trials assessing the impact of KDs compared to mixed macronutrient control (CON) diets on body composition in healthy (without chronic disease), physically active men or women ≥18 years were included in the current analysis. Dietary carbohydrate must have been < 50 g/d or ketosis confirmed by measurement of circulating β-hydroxybutyrate (βhb) concentration ≥ 0.5 mmol/L. Diet must have been consumed for ≥14 days.

### Exclusion criteria

Studies examining the effect of a KD on sedentary populations, animal models, or children/adolescents (< 18 years) were excluded. Studies were excluded if carbohydrate intake was > 50 g/d and ketosis was not confirmed by measurement of circulating βhb concentration > 0.5 mmol/L. If diets were administered for < 14 days, or very low calorie (< 1200 kcal/day) KD was used the study was excluded. Studies that did not assess body composition were excluded.

### Bias and limitations

A bias analysis was performed by JLC in accordance with PRISMA guidelines recommend by Sterne et al. [21]. Ratings including low, some, or high concern of randomization, intervention, outcomes, and reporting bias were assigned to each study.

### Data extraction

Data were extracted from the 13 articles that met the established inclusion criteria. Studies were identified as being crossover/longitudinal [22–25], or parallel [11, 15, 16, 26–31] designs. Sex, age, weight, body mass index (BMI), and population description (i.e. recreationally active, endurance athlete, etc.) were extracted to provide volunteer descriptive characteristics. Total energy intake was extracted as kcal/d. Dietary fat, carbohydrate, and protein intake data were extracted and presented as percent energy intake. Methods for prescribing diet (i.e. counseling, controlled feed, etc.) and obtaining body composition data (i.e. dual energy x-ray absorptiometry (DXA) bio-electrical impedance analysis (BIA), etc.) were extracted from each study to account for variabilities in intake outcomes and measurement methods. Pre- and post- dietary intervention body mass and composition data were extracted from each study and reported as the Delta (∆; post - pre). If a manuscript only provided partial outcomes (i.e. FM or FFM), researchers subtracted from the total to get the other component. Data that were not reported numerically were generated from provided figures by digitally measuring the height of the histogram bar and calculating relative to measured y-axis units [32]. Energy balance was calculated from changes in FM and FFM from Post minus Pre for KD and CON as [33]: Energy Balance = ((∆FM g × 9.51 kcal/g) + (∆FFM g × (1–0.73) × 4.40 kcal/g))/days where 0.73 represents the estimated aqueous fraction of FFM [34] and days is the duration participants consumed the study diets. It should be noted that FFM hydration can vary between individuals [35], athletic populations [36], and those following KD versus a non-KD [37]. Assuming FFM hydration of 0.73 may result in a systematic error that limits the accuracy of energy balance estimation.

## Results

### Study characteristics

Of the 13 studies identified, 9 employed parallel design and 4 crossover/longitudinal design (Fig. 1). Within these 13 studies, 243 individuals participated (parallel: 196; 129 men, 67 women, and crossover: 47; 22 men, 25 women) (Table 1). Mean dietary intervention duration was 61 (range: 56–84 parallel, 21–84 crossover/longitudinal) days.

**Table 1:** Volunteer characteristics

First author, ref	N	Population description	Sex	Age (yr)	Wt (kg)	BMI
***Crossover Study Design***						
Greene et al. [22],	12	Elite powerlifting and Olympic weightlifting	7 M, 5F	35 ± 11	78 ± 12	NR
Heatherly et al. [24], ^L^	8	Trained runners	M	40 ± 10	82 ± 7	NR
Nazaraweicz et al. [25],^L^	20	Recreationally active	F	21-23^*R*^	62 ± 10	21.5 ± 2.1
Prins et al. [23],	7	Recreational distance runners	M	36 ± 8	69 ± 2	21.5 ± 1.1
***Parallel Study Design***						
Dostal et al. [26],	KD: 12 CON: 12	Recreationally active	7 M, 17F	25 ± 2 24 ± 4	67 ± 10 73 ± 15	NR
Gregory et al. [30],	KD: 12 CON: 15	Recreational CrossFit trainees	5 M, 22F	35 ± 9 34 ± 9	77 ± 14 72 ± 13	26.0 ± 2.9 26.0 ± 2.8
Kephart et al. [28],	KD: 7 CON: 5	Recreational CrossFit trainees	9 M, 3F	32 ± 8 29 ± 7	83 ± 22 77 ± 12	28.3^a^ 26.6^a^
LaFountain et al. [29],	KD: 15 CON: 14	Military personnel	25 M, 4F	27 ± 7 25 ± 9	86 ± 8 80 ± 6	27.9 ± 2.9 24.9 ± 2.4
McSwiney et al. [16],	KD: 9 CON: 11	Endurance athletes	M	34 ± 7 32 ± 6	86 ± 14 77 ± 10	25.6 ± 3.0 23.9 ± 2.9
Paoli et al. [31],	KD: 9 CON: 10	Competitive body builders	M	26 ± 5 31 ± 10	86 ± 15 89 ± 11	26.9 ± 1.9 26.6 ± 2.0
Vargas et al. [15],	KD: 9 CON: 10	Resistance-trained	M	28 ± 4 27 ± 6	79 ± 8 75 ± 5	24.4 ± 2.6 23.9 ± 1.6
Vargas-Molina et al. [27],	KD: 10 CON: 11	Resistance-trained	F	27 ± 4 28 ± 4	62 ± 10 63 ± 6	23.8 ± 3.6 23.7 ± 2.2
Wilson et al. [11],	KD: 13 CON: 12	Resistance-trained	M	24 ± 5 21 ± 4	80 ± 15 78 ± 10	NR

Values mean ± SD

Abbreviations: *NR* not reported

^L^indicates longitudinal study design

^*R*^indicates range

^a^indicates BMI calculated from available data

### Macronutrient intake and body composition

Mean energy and relative macronutrient intake during parallel and crossover/longitudinal studies for KD was 2318 kcals, 8% carbohydrate, 70% fat, 22% protein, and for CON was 2433 kcals, 52% carbohydrate, 29% fat, and 19% protein (Table 2). Body mass decreased 2.7 kg in KD, and increased 0.3 kg in CON (Table 3). Fat mass decreased 2.3 kg in KD and 0.3 kg in CON. Fat-free mass decreased 0.3 kg in KD and increased 0.7 kg in CON. Based on changes in body composition, calculated energy balance was − 339 kcal/d in KD and 5 kcal/d in CON. Calculations from Heatherly et al. [24] and Nazaraweicz et al. [25] were excluded from KD energy balance average to account for unreported data in CON.

**Table 2:** Study Description

First author, ref	Body composition measurement method	Diet prescription method	Diet	Dietary intake				Energy Intake Intent
				Kcal/d	CHO % (g/kg)	FAT % (g/kg)	PRO % (g/kg)	
***Crossover Study Design***								
Greene et al. [22],	DXA	Counseling and education materials	KD CON	2072 2058	8 (0.5) 45 (2.9)	69 (2.0) 33 (1.0)	23 (1.5) 22 (1.5)	Balance
Heatherly et al. [24], ^L^	BIA	Education materials	KD CON	1885^a^ 2769^a^	6^a^(0.3) 45^a^(3.9)	64^a^(1.6) 39^a^(1.5)	28^a^ (1.6) 16^a^(1.6)	Balance
Nazaraweicz et al. [25],^L^	BIA	Education materials	KD CON	NR NR	13 NR	74 NR	13 NR	Deficit
Prins et al. [23],	BIA	Counseling and education materials	KD CON	2947 2837	6 (0.6) 56 (5.1)	69 (3.2) 28 (1.3)	25 (2.6) 16 (1.7)	Balance
***Parallel Study Design***								
Dostal et al. [26],	BIA	Counseling and education materials	KD CON	1962 1783	8 (0.6) 45 (2.7)	69 (2.2) 34 (0.9)	23 (1.7) 18 (1.1)	Balance
Gregory et al. [30],	DXA	Education materials	KD CON	1580 1746	11^a^(0.5) 43^a^(2.3)	65^a^(1.3) 38^a^(1.2)	23^a^(1.2) 19^a^(1.2)	Balance
Kephart et al. [28],	DXA	Education materials	KD CON	1948 NR	3^a^(0.2) NR	79^a^(2.0) NR	18^a^(1.1) NR	Balance
LaFountain et al. [29],	DXA	Counseling	KD CON	NR NR	NR NR	NR NR	NR NR	Balance
McSwiney et al. [16],	DXA	Counseling and education materials	KD CON	3022 2644	6^a^(0.5) 65^a^(5.6)	77^a^(3.0) 20^a^(0.8)	17^a^(1.5) 15^a^(1.3)	Balance
Paoli et al. [31],	BIA	Counseling	KD CON	3443 3529	5 (0.5) 55 (5.5)	68 (3.0) 20 (0.9)	27 (2.7) 25 (2.5)	Balance
Vargas et al. [15],	DXA	Controlled feeding	KD CON	NR NR	10 55	70 25	20 20	Excess
Vargas-Molina et al. [27],	DXA	Counseling	KD CON	1710 1980	9 (0.7) 57 (4.5)	64 (2.0) 23 (0.8)	27 (1.9) 20 (1.6)	Balance
Wilson et al. [11],	DXA	Counseling	KD CON	2609 2550	5^a^(0.4) 50^a^(4.1)	75^a^(2.7) 29^a^(1.0)	20^a^(1.6) 21^a^(1.7)	Balance

Data are reported as mean

Abbreviations: *DXA* Dual-energy X-ray absorptiometry, *BIA* bio-electrical impedance analysis, *NR* not reported

^L^indicates longitudinal study design

^a^indicates percentages calculated from available data

**Table 3:** Change in body mass and composition on ketogenic or control diets

First author, ref	Duration (d)	KD				CON			
		Body Mass (kg)	Fat Mass (kg)	Fat Free Mass (kg)	Calculated Energy Balance (kcal/d)	Body Mass (kg)	Fat Mass (kg)	Fat Free Mass (kg)	Calculated Energy Balance (kcal/d)
Dostal et al. [26],	84	−3.6	− 2.9	− 0.7^a^	− 338	− 0.9	− 0.4	− 0.5^a^	− 52
Greene et al. [22],	84	− 1.7	0	−1.7	− 24	1.6	1.1	0.5	132
Gregory et al. [30],	42	− 3.5	− 2.8	− 0.4	−645	0.2	0.1	0.1	25
Heatherly et al. [24], ^L^	21	−2.1	− 2.2	−0.1	− 1002	–	–	–	–
Kephart et al. [28],	84	−3	− 2.5	− 0.5	− 290	− 0.3	− 0.3	0	−34
LaFountain et al. [29],	84	− 7.7	−5.9	− 1.8^a^	− 693	0.1	− 0.6	0.7^a^	− 58
McSwiney et al. [16],	84	− 5.9	−4.6	0.3	− 517	− 0.8	− 0.5	0.1	−55
Nazaraweicz et al. [25],^L^	28	− 1.7	− 1.7^a^	0.0^a^	− 577	–	–	–	–
Paoli et al. [31],	56	− 0.8	− 1.4	0.6	− 250	1.3	− 0.9	2.2	202
Prins et al. [23],b	42	0.5	−0.2	0.4	−34	0.1	0.5	−0.4	102
Vargas et al. [15],	56	−1.4	− 1.1	− 0.3	− 193	0.9	− 0.4	1.4	−38
Vargas-Molina et al. [27],	56	−2.2	− 1.1	−0.7	− 202	0.8	0.4	0.7	83
Wilson et al. [11],	70	−2.6	− 4.2	1.5	− 545	0.6	− 2.2	2.7	− 253

^L^indicated longitudinal study design

^a^indicates values calculated from available data

^b^Delta calculated as day 42 – day 4

### Risk Bias

A risk of bias assessment for the individual studies included in this systematic review are reported in Supplementary Table 2. Overall, risk of bias identified some concern of bias. This level of bias was due to use of longitudinal study design, self-selection of dietary intervention group, and inability to blind participants to the study intervention.

## Discussion

### Body mass

The primary findings from the aggregated results of the 13 studies identified in this systematic review show that body mass declines when healthy, physically active individuals consume a KD. This observation is particularly compelling, since only 1 of those 13 studies intentionally placed participants on a hypocaloric diet. Participants in the 12 other studies ate ad libitum*.* Therefore, the mechanism by which consuming a KD results in loss of body mass is not obvious.

One possible explanation for the loss of body mass with KD is that the participants in those 12 studies in which energy restriction was not prescribed, were in fact, consuming a hypocaloric diet, despite ad libitum intake. Results from Vargas-Molina et al. [27], and Gregory et al. [30] indicated that energy intake of participants consuming KD was below the prescribed amount, whereas CON participants consumed the prescribed energy intake to maintain energy balance. Using food records to assess dietary intake, Vargas-Molina et al. [27], and Gregory et al. [30] reported energy intake being of 270 kcal and 420 kcal/d lower when consuming KD compared to CON. Lower energy intake with KD in these studies align with calculated energy balance of − 202 kcal/d, and − 645 kcal/d determined by changes in body composition in the current systematic review. Furthermore, body mass decreased by 2.2 kg in KD versus a 0.8 kg increase for CON in Vargas-Molina et al. [27], and Gregory et al. [30] similarly saw a decrease of 3.5 kg in KD and 0.2 kg increase in CON, offering more confirmative evidence that energy deficits occurred in KD, while CON maintained energy balance. The highly restrictive nature of KD may have limited the overall energy consumption of participants due to reductions in food choice, or a potential dislike in foods available to consume. On the other hand, self-reported dietary intake in 5 studies included in this review indicated energy intake for participants following KD was equivalent or greater than CON participants, yet the KD participants still lost body mass. However, reliance on self-reported food logs or 24 h recalls to obtain dietary intake typically results in underreporting [38]. Thus, the lack of differences in energy intake observed between groups may simply reflect food diary or recall errors rather than true energy deficit, which may be better reflected in loss of body mass or calculated energy balance as indicated in Vargas Molina et al. [27] and Gregory et al. [30].

There is also evidence that suggests consumption of KD increases satiety and decreases appetite, which could aid in body mass reduction. Surveys of self-reported weight loss symptoms administered to participants consuming KD or low-fat, energy restricted diets (< 30% kcal from fat) for 6 months found KD were more likely to report significant improvements in hunger compared to low-fat, energy restricted diets [39]. Along with improvements in feeling of hunger, body mass declined 12.9 kg in KD compared to 6.7 kg in low-fat, energy-restricted diets [39]. McClernon et al. [39] attributed these findings to kcal reduction of one macronutrient (carbohydrate) versus kcal reduction of all macronutrients, which could reduce food cravings. Though KD has restrictions, avoidance of one macronutrient may be easier for free living individuals to understand and follow. Similarly, in a short-term, 2 week study conducted on obese patients with type II diabetes, despite energy intake decreasing from 3111 kcal/d to 2164 kcal/d self-reported ratings of hunger and satisfaction did not change when consuming KD compared to habitual diets [40]. Boden et al. [40] suggested the potential mechanism resulting in reduced appetite was decreased concentrations in circulating insulin, as some studies have indicated insulin may increase food intake [41, 42], a finding that has recently been questioned [43]. However, since satiety, appetite, or hunger were not assessed in the individual studies included in this systematic review, it is unclear if alterations in the outcomes influenced decreases in body mass when consuming KD.

Alternatively, KD may cause an increase in daily energy expenditure. Several studies have reported increased oxygen uptake during higher intensity (> 70% VO2max) exercise compared to CON, suggesting reductions in exercise economy may elevate exercise-induced energy expenditures [44–48]. The mechanism causing reduced exercise economy and increased energy expenditure following KD is unclear. It has been speculated that increased oxygen uptake during exercise might be the result of changes in proton gradient uncoupling in mitochondria at the skeletal muscle level resulting in reduced energy production relative to the oxidative cost [45]. Another proposed mechanism is alterations in the microbiome while consuming KD. Murtaza et al. [49] reported significant shifts in elite race walkers microbiome, which were associated with exercise economy following 21 days on a KD. Regardless of the cause, if exercise-induced energy expenditure is increased when consuming KD, this would contribute to an increased total daily energy expenditure, and the magnitude of that effect and the associated loss of body mass might be pronounced in participants engaged in vigorous training programs. With appropriate adaptation time, maintenance of physical performance can be met in vigorous training programs while consuming KD [50].

### Fat mass

The overall reductions in body mass following KD appears to be primarily accounted for by losses in FM. Those losses of FM may be mediated by alterations in substrate oxidation facilitating fat oxidation as an energy source while adhering to KD. Increased fat and decreased carbohydrate intake when consuming KD increases concentrations of free-fatty acids (FFA) in circulation, while reducing circulating glucose and insulin concentrations stimulating lipolysis [51, 52]. Alterations in substrate availability and hormonal responses to KD result in increased uptake FFA in the liver where they undergo β-oxidation to produce acetyl-CoA (Ac-CoA). Ketone bodies, acetoacetate (AcAc), acetone, and βhb, are then derived from Ac-CoA [53, 54]. Sequential reactions of Ac-CoA molecules to acetoacetyle-CoA (AcAc-CoA) catalyzed by Ac-CoA acetyltransferase (ACAT), generate hydroxymethylglutaryl-CoA (HMG-CoA) catalyzed by hydroxymethylglutaryl CoA synthase (HMGCS). HMG-CoA is then converted to AcAc by HMG-CoA lyase (HMGCL). Some AcAc enters circulation where it can be further reduced to acetone, while most is reduced to βhb by β-hydroxybutyrate dehydrogenase (BDH). Ketone bodies can then be transported out of circulation into peripheral tissue by monocarboxylate transporters (MCTs) where ketolytic enzymes BDH, acetoacetyle-CoA thiolase (ACAT), and succinyl-CoA/3-ketoacid CoA transferase convert ketone bodies to Ac-CoA to enter the tricarboxylic acid (TCA) cycle to be oxidized for energy [50, 53, 55, 56].

Increased fatty acid and decreased carbohydrate availability for fuel use during exercise results in drastic shifts in substrate oxidation when consuming KD compared to mixed macronutrient control diets (CON) [13, 44]. Specifically, peak whole-body fat oxidation in athletes consuming KD is ~ 1.2–1.6 g/min during exercise, compared to ~ 0.6 g /min in athletes consuming CON [13, 14, 57]. The ≥2 fold increase in whole-body fat oxidation while consuming KD is likely the result of increased fat availability in skeletal muscle, stored as intramuscular triglycerides [58], as well as alterations in molecular signaling pathways which regulate the uptake and oxidation of fatty acids [59]. Consumption of high-fat, low-carbohydrate diets increases fatty acid translocase (FAT/CD36), a scavenger receptor on cell surface that imports fatty acids inside cells, and fatty acid binding protein (FABP) to transport fatty acids from the extracellular to intracellular membrane of the cell [60, 61]. Increase of fatty acid transport into the cell increases activity of long-chain acyl-CoA synthetase (ACSL1) to convert fatty acids to fatty acyl-CoA to be taken up into the mitochondria via carnitine palmitoyl transferase 1a (CPT1a) for β-oxidation and generation of Ac-CoA which enters the TCA cycle for energy production [59, 61]. Expression and protein content of these key regulators of fatty acid uptake, transport, and oxidation are governed by peroxisome proliferator-activated receptors (PPARs) transcription factors [62]. The expression of PPARs are upregulated in response to low carbohydrate availability to increase the expression of FAT/CD36, CPT1a, hydroxyacyl-CoA dehydrogenase (HADHA), and long-chain acyl-CoA dehydrogenase (LCAD) expression in skeletal muscle to increase the rate of β-oxidation [63, 64]. Metabolic and molecular alterations associated with consumption of KD may contribute to potential favorable changes in body composition.

The studies included in this review which measured substrate oxidation all reported shifts that were indicative of increased fat oxidation at rest [28, 29] or during exercise under fed [16] or fasted conditions [23, 24]. For example, Kephart et al. [28] and LaFountain et al. [29], reported ~ 10% reductions in respiratory exchange ratio (RER) following an overnight fast in participants consuming a KD compared to CON. LaFountain et al. [29], calculated that fat constituted 84% of total non-protein derived energy in KD compared to 54% in CON. Similarly, McSwiney et al. [16] reported RER was significantly lower throughout the duration of 100 km time trial (TT) in KD compared to CON. Heatherly et al. [24] observed peak fat oxidation was 0.81 g/min in KD during fasted 5-km time trail (TT) compared to 0.29 g/min in CON, and Prins et al. [23], similarly observed peak fat oxidation was 0.78 g/min in KD compared to 0.17 g/min in CON during submaximal 5-km TT. Together these data indicate that shifts in substrate oxidation are not only reflective of changes in exogenous fat oxidation, but endogenous fat oxidation as well.

Reductions in FM with KD appear to be in conflict with data suggesting increases in circulating ketone bodies are anti-lipolytic [54, 56, 65]. Using infusion protocols or oral ketone supplements to induce acute ketosis, circulating concentrations of FFA and glycerol are decreased compared to placebo [66–68]. Animal models have indicated that lower FFA concentrations with ketosis are due to inhibited adipocyte lipolysis [69, 70]. However, dietary modification (< 50 g/d carbohydrate, ~ 70–80% kcal from fat) to induce ketosis results in hormonal responses that induce lipolysis. Langfort et al. [51, 52] reported consumption of KD resulted in higher circulating concentrations of pro-lipolytic hormones epinephrine and norepinephrine, and lower concentrations of the anti-lipolytic hormone insulin compared to CON. Hormonal shifts while consuming KD resulted in higher concentrations of FFA and fat oxidation during exercise compared to CON [51, 52]. Discrepancies between supplemental and diet induced ketosis indicate unique metabolic responses to each intervention [71]. Inhibition of lipolysis with rapid elevations in circulating ketone body concentrations with infusion protocols or oral supplementation suggest some negative feedback mechanism to suppress endogenous ketogenesis. Stimulation of lipolysis during more moderate prolonged ketosis when consuming KD indicates metabolic adaptations which favor endogenous ketogensis. Hormonal responses to KD support the mobilization of endogenous fat stores for fuel use, and may, in part, contribute to decreases in FM when consuming KD.

### Fat-free mass

It appears FFM is maintained while consuming KD. Collectively, only 0.3 kg FFM was lost during KD despite a 2.9 kg loss of total body mass. Preservation of FFM despite reductions in body mass may be the result of moderate to high amounts of protein (~ 1.2–2.0 g/kg/d) consumption associated with KD [12]. During periods of weight loss due to energy restriction there is increased reliance on amino acids for energy production and gluconeogenesis [27], and increased whole-body proteolysis creates a negative protein balance, resulting in the loss of FFM [72]. However, if dietary protein consumption during energy restriction is at least 1.6 g/kg/d, double the current RDA, protein balance is less negative and FFM is spared during periods compared to lower protein diets [10, 73, 74]. In agreement with these past findings, average protein intake of studies included in the current systematic review was ~ 1.7 g/kg/d, ranging from 1.1–2.7 g/kg/d [11, 15, 22–24, 26–29, 31]. Thus, sufficient dietary protein may be consumed while consuming KD to maintain muscle protein synthetic rates [75, 76] and/or minimized whole-body protein breakdown [77], attenuating declines in FFM despite decreasing body mass.

Elevations in the ketone body βhb may also contribute to maintenance of FFM despite reductions in body mass when consuming KD. Vandoorne et al. [78] reported that when a ketone ester supplement was consumed during recovery from exercise to elevate circulating βhb concentrations to ~ 3.0 mmol/L, phosphorylation of two downstream targets of mTORC1, p70S6K and 4E-BP1, in skeletal muscle was greater than placebo, indicating greater anabolic signaling. That in vivo observation was confirmed by in vitro analysis in which C2C12 myotubes were incubated in ketone bodies βhb, AcAc and leucine together or separately [78]. Similar to the human study, phosphorylation of p70S6K and 4E-BP1 increased in vitro when myotubes were incubated with ketone bodies alone or in combination with leucine [78]. Ketone bodies were also shown to increase muscle protein synthesis, estimated from puromycin, when combined with 1.5 mmol/L of leucine to a similar extent of 5.0 mmol/L of leucine alone [78]. Thomsen et al. reported infusion of βhb to achieve a concentration of 3.5 mmol/L in healthy men, result in higher skeletal muscle protein balance, as determined by phenylalanine trace infusion methodologies, during acute inflammation compared to placebo. Higher protein balance with βhb infusion were the result of reduced phenylalanine rate of disappearance (e.g., synthesis) and appearance (e.g., breakdown), with reduction of protein breakdown exceeding reductions in synthesis compared to placebo. Collectively, these data indicate βhb may have a function in altering skeletal muscle synthesis and breakdown. However, as described in the section above, there are differences in the metabolic response to infusion protocols or oral supplementation compared to KDs. The impact that elevations of βhb have on protein synthesis and breakdown while consuming KD remain unclear.

Despite the collective finding of maintenance of FFM when consuming KD, the responses reported in the literature reviewed exhibit a great deal of heterogeneity, with individual studies reporting a decrease [22, 27, 29], increase [11], or no change [16, 23–26, 28, 31] compared to baseline. This variability may be attributable to differences in severity of weight loss, study design, and population. More severe energy deficits, resulting in greater weight loss are associated with greater loss in FFM [79]. For example, FFM decreased 1.8 kg in the study by LaFountain et al. [29] when participants consuming KD lost 7.7 kg body mass, whereas FFM was maintained in work by Vargas et al. [15], where participants lost only 1.4 kg body mass. Inclusion of resistance training programs during consumption of KD may also facilitate FFM maintenance in most studies with moderate weight loss [11, 31]. Wilson et al. [11] reported college males familiar with resistance training gained 1.5 kg FFM despite reductions in body mass when consuming KD for 70 days. The progressive resistance exercise training model along with participants being only recreationally active may have contributed to facilitating gains in FFM with KD. A meta-analysis by Ashtary-Larky et al. [80] provides a thorough review of the literature discussing the effects of resistance training with KD. Regardless, heterogeneity across studies appears to be greatest in the changes observed in FFM with KD suggesting that further investigation to understand the impact of KD on FFM is warranted.

### Risk of Bias

There are several limitations to the findings of this systematic review. The risk-of-bias analysis identified that there is overall some risk-of-bias in the literature. Bias across these studies is the result of allowing participants to self-select diet interventions, and inability to blind participants to their treatment group, even with randomization. In addition, the majority of studies included in this manuscript relied on counseling and education materials to prescribe study diets rather than investigator-controlled diets. Participants knowing their treatment groups and not following a controlled diet, may have influenced eating behavior, potentially contributing to the reductions in body mass when consuming KD. Furthermore, several studies used BIA to measure longitudinal changes in body composition. While this method is generally reliable for cross-sectional analysis, it is less reliable for longitudinal, repeated measurements [81]. Finally, some investigations did not report all body composition components or dietary intake data. Lack of complete data precluded a meta-analysis to be conducted to statistically compare differences in outcome variables and heterogeneity between KD and CON. Additionally, lack of complete data being published increases risk-of-bias of the individual publications and required some assumptions being made in the current systematic review.

## Conclusion

In conclusion, the overall results from individual studies indicated that body mass is reduced when consuming KD. Reductions in body mass appear to be due to negative energy balance, whether intentional or not, as 12 of the 13 studies included in this review aimed to maintain energy balance while participants consumed KD. Decreased body mass was primarily driven be decreases in FM, with FFM maintained when consuming KD. For athletes looking to optimize their strength/power to mass ratio, KD may promote a more rapid weight loss by mobilizing fat stores while retaining FFM. However, there is some risk-of-bias and limitations in in the literature due to self-selection of diet intervention, lack of controlled feeding, and use of BIA to measure composition. To avoid these potential biases and limitations, future investigations should randomize participants into treatment groups, provide all food and drinks to ensure desired energy and macronutrient intakes, and use more sensitive techniques to measure body composition.

## Supplementary Information

**Additional file 1: Supplemental Table 1**. Search terms.

**Additional file 2: Supplemental Table 2.** Risk of bias for publications included in systematic review.

## Data Availability

All extracted data are presented in this manuscript. The corresponding author may be contacted for any data requests or questions.

## Abbreviations

AcAc: Acetoacetate
AcAc-CoA: Acetoacetyle-CoA
ACAT: Acetoacetyle-CoA thiolase
Ac-CoA: Acetyl-CoA
ACAT: Ac-CoA acetyltransferase
βhb: β-hydroxybutyrate
BDH: β-hydroxybutyrate dehydrogenase
BIA: Bio-electrical impedance analysis
BMI: Body mass index
CPT1a: Carnitine palmitoyl transferase 1a
DXA: Dual energy x-ray absorptiometry
FM: Fat mass
FFM: Fat-free mass
FABP: Fatty acid binding protein
FAT/CD36: Fatty acid translocase
FFA: Free fatty acid
HMGCL: HMG-CoA lyase
HADHA: Hydroxyacyl-CoA dehydrogenase
HMG-CoA: Hydroxymethylglutaryl-CoA
HMGCS: Hydroxymethylglutaryl CoA synthase
KD: Ketogenic diet
LCAD: Long-chain acyl-CoA dehydrogenase
ACSL1: Long-chain acyl-CoA synthetase
CON: Mixed macronutrient control diets
MCTs: Monocarboxylate transporters
PPARs: Peroxisome proliferator activated receptors
PRISMA: Preferred Reporting Items for Systematic Reviews and Meta-Analyses
RER: Respiratory exchange ratio
RDA: Recommended Dietary Allowance
TT: Time trail
TCA: Tricarboxylic acid cycle
