# Supplementary material for: Body composition changes in physically active individuals consuming ketogenic diets: a systematic review
Source: J Int Soc Sports Nutr. 2021 Jun 5;18:41. doi: 10.1186/s12970-021-00440-6 (PMC8180141; doi:10.1186/s12970-021-00440-6)
Supplement: Supplementary file 1 — Additional file 1: Supplemental Table 1. Search terms. [file 12970_2021_440_MOESM1_ESM.docx]

**Supplemental Table 1**: Search terms

| Intervention Terms | Outcome Terms | Population Terms | Exclusion Criteria |
| --- | --- | --- | --- |
| “Ketogenic Diet” | “Fat-Free Mass” | Humans | Rats |
| Ketosis | “Fat Mass” | Men | Mice |
|  | “Body Composition” | Women | Pigs |
|  | “Body Weight” | Athletes | Dogs |
|  | “Lean Body Mass” | Runners | Sheep |
|  | “Muscle Mass” | Rowers | Horse |
|  |  | Cyclists |  |
|  |  | “Power lifters” |  |
|  |  | “Body building” |  |
|  |  | Crossfit |  |

Search terms used in Pubmed (<http://www.ncbi.nlm.nih.gov/pubmed>) and Cochrane Library (<https://www.cochranelibrary.com/>) to capture relevant articles. Searches took place on 24 March 2020 and 20 April 2020, respectively, and were not restricted by publication date.

Search terms as they appear in search Pubmed search engine:

((((("ketogenic diet") OR Ketosis)) AND (((((("fat-free mass") OR "fat mass") OR "body composition") OR "body weight") OR "lean body mass") OR "muscle mass")) AND ((((((((((humans) OR men) OR women) OR athletes) OR runners) OR rowers) OR cyclists) OR "power lifters") OR "body building") OR crossfit)) NOT ((((((rats) OR mice) OR pigs) OR dogs) OR sheep) OR horse)

Search terms as they appear in Cochrane Library search engine:

(ketogenic diet OR ketosis) AND (“fat-free mass” OR “fat mass” OR “body composition” OR “body weight” OR “lean body mass” OR “muscle mass”) AND (humans OR men OR women OR athletes OR runners OR rowers OR cyclists OR “power lifters” OR “body building” OR crossfit) NOT (rats OR mice OR pigs OR dogs OR sheep OR horse)
