# Supplementary material for: Body composition changes in physically active individuals consuming ketogenic diets: a systematic review
Source: J Int Soc Sports Nutr. 2021 Jun 5;18:41. doi: 10.1186/s12970-021-00440-6 (PMC8180141; doi:10.1186/s12970-021-00440-6)
Supplement: Supplementary file 2 — Additional file 2: Supplemental Table 2. Risk of bias for publications included in systematic review. [file 12970_2021_440_MOESM2_ESM.docx]

Online Supplementary Data

**Supplemental Table 2:** Risk of bias for publications included in systematic review

| Reference | Bias arising from the randomization process | Bias due to deviations from intended interventions | Bias due to missing outcome data | Bias in measurement of the outcome | Bias in selection of the reported result | Overall risk of bias |
| --- | --- | --- | --- | --- | --- | --- |
| ***Crossover Study Design*** |  |  |  |  |  |  |
| Greene et al., (22) | L | H | L | S | L | L |
| Heatherly et al., (24)^L^ | H | H | L | H | L | H |
| Nazaraweicz et al., (25)^L^ | H | H | H | H | L | H |
| Prins et al., (23) | L | H | L | S | L | L |
| ***Parallel Study Design*** |  |  |  |  |  |  |
| Dostal et al., (26) | H | H | L | S | L | S |
| Gregory et al., (30) | L | H | L | S | L | L |
| Kephart et al., (28) | H | H | H | H | L | H |
| LaFountain et al., (29) | H | H | S | S | L | S |
| McSwiney et al., (16) | H | H | L | S | L | S |
| Paoli et al., (31) | L | H | L | S | L | L |
| Vargas et al., (15) | L | H | H | S | L | S |
| Vargas-Molina et al., (27) | L | H | L | S | L | L |
| Wilson et al., (11) | L | H | L | S | L | L |
| Overall Risk of Bias | S | H | L | S | L | S |
| Abbreviations: H = high concern, L = low concern, S = some concern, ^L^ = longitudinal study design | | | | | | |
